# Diagnosis and treatment of thrombotic microangiopathy

**DOI:** 10.1111/ijlh.13954

**Published:** 2022-09-08

**Authors:** Gemma L. Thompson, David Kavanagh

**Affiliations:** ^1^ Complement Therapeutics Research Group, Translational and Clinical Research Institute Newcastle University Newcastle upon Tyne UK; ^2^ National Renal Complement Therapeutics Centre Royal Victoria Infirmary Newcastle upon Tyne UK

**Keywords:** haemolytic uraemic syndrome, STEC‐HUS, thrombotic microangiopathy, thrombotic thrombocytopenic purpura

## Abstract

Thrombotic microangiopathy (TMA) is characterized by thrombocytopenia, microangiopathic haemolytic anaemia and end organ damage. TMAs have varying underlying pathophysiology and can therefore present with an array of clinical presentations. Renal involvement is common as the kidney is particularly susceptible to the endothelial damage and microvascular occlusion. TMAs require rapid assessment, diagnosis, and commencement of appropriate treatment due to the high morbidity and mortality associated with them. Ground‐breaking research into the pathogenesis of TMAs over the past 20 years has driven the successful development of targeted therapeutics revolutionizing patient outcomes. This review outlines the clinical presentations, pathogenesis, diagnostic tests and treatments for TMAs.

## 1 INTRODUCTION

Thrombotic microangiopathy (TMA) describes a pathological state where vessels are occluded by platelet rich thrombi leading to thrombocytopenia and microangiopathic haemolytic anaemia (MAHA).^1^ Depending upon the cause of the TMA, thrombi can be systemic, or more commonly intrarenal, and inevitably lead to end organ damage.

**FIGURE 1:**
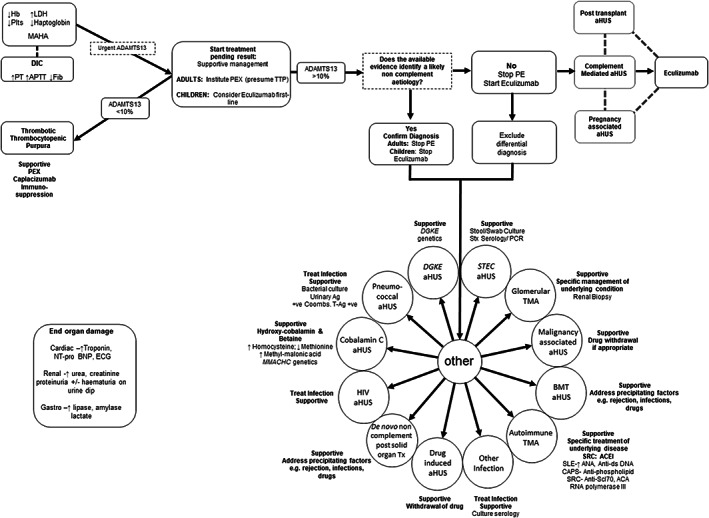
Diagnostic and treatment algorithm for thrombotic microangiopathy (TMA). In a patient presenting with MAHA and thrombocytopenia suggestive of a TMA a thorough diagnostic evaluation will usually reveal the underlying aetiology and guide treatment. As many of the investigations will not be available at initial presentation the initial focus should be on the consideration of TTP given the high mortality if untreated. In adults PE should be instituted on the presumption that it is TTP unless other evidence is available that strongly suggests an alternative aetiology. In children, in whom TTP is rarer, first‐line treatment with eculizumab should be considered if complement‐mediated aHUS is suspected and should not be delayed whilst ADAMTS13 activity is determined. In the absence of a defined aetiology, complement‐mediated aHUS is presumed and treatment with eculizumab is recommended pending the complete evaluation. +ve, positive; Ab, antibody; ACA, anti‐centromere antibody; ACEI, angiotensin converting enzyme inhibitor; ADAMTS13, a disintegrin and metalloproteinase with a thrombospondin type 1 motif, member 13; Ag, antigen; aHUS, atypical haemolytic uraemic syndrome; AKI, acute kidney injury; ANA antinuclear antibody; Anti‐ds DNA, anti‐double stranded DNA; anti‐Scl‐70, anti‐topoisomerase I antibody; BMT; bone marrow transplant; CAPS, catastrophic antiphospholipid syndrome; CMV, cytomegalovirus; DIC, disseminated intravascular coagulation; FACS, flow cytometry; FH, factor H; FI, factor I; Hb, haemoglobin; HIV, human immunodeficiency virus; HUS, haemolytic uraemic syndrome; LDH, lactate dehydrogenase; MAHA, microangiopathic haemolytic anaemia; MLPA, multiplex ligation‐dependent probe amplification; MMA, methylmalonic acid; Nt‐proBNP; brain natriuretic peptide PCR, polymerase chain reaction; PE, plasma exchange; SLE, systemic lupus erythematosus; SRC, scleroderma renal crisis; STEC, Shiga toxin producing *Escherichia coli*; Stx, Shiga toxin; T‐Ag, Thomsen‐Friedenreich antigen; TMA, thrombotic microangiopathy; TTP, thrombotic thrombocytopenic purpura

The clinical presentation of TMA can vary, and the differential diagnosis is wide, therefore laboratory tests are important alongside a thorough history and examination. Thrombocytopenia, MAHA, and end organ damage are the common elements of all TMAs. Thrombocytopenia is due to platelet aggregation and thrombi formation. MAHA is caused by red blood cell fragmentation in the microvasculature, with schistocytes seen on peripheral blood film. Lactate dehydrogenase (LDH) is raised due to tissue ischemia and cell lysis. Low plasma haptoglobin is a marker of haemolysis as it binds to free haemoglobin and the complex is cleared by macrophages. Coombs test is generally negative apart from in pneumococcal haemolytic uraemic syndrome (Figure 1). Coagulation screen is normal in TMA in contrast to elevated PT and aPTT and low fibrinogen in disseminated intravascular coagulation.

Renal involvement is common to most TMAs due to its vulnerability to occlusion and endothelial damage. Extra renal manifestations can occur in each TMA and do not always help with the identification of the aetiology.^1^

TMAs can have a high mortality rate. Rapid diagnosis and treatment are key to the survival and quality of life for the patients. In this review, we will discuss the clinical presentation, diagnosis, and treatment of the different clinical types of TMAs.

## 2 THROMBOTIC THROMBOCYTOPENIC PURPURA INCIDENCE AND PATHOGENESIS

Thrombotic thrombocytopenic purpura (TTP) is a rare systemic form of TMA due to a severe deficiency in ADAMTS13. ADAMTS13 is an enzyme (a disintegrin and metalloprotease with thrombospondin type 1 motif 13), which cleaves von Willebrand factor (VWF).^2^ Deficiency of ADAMTS13 in TTP results in the formation of ultra large VWF multimers on the endothelium. Platelet attachment to these ultra large multimers occurs in high shear conditions, which unravel VWF and expose the platelet binding site. This leads to platelet adhesion, aggregation and ultimately the formation of microvascular thrombi.

TTP is rare in the United Kingdom with an annual incidence of six per million.^3^ TTP may be either acquired (aTTP) or congenital (cTTP). aTTP is the common form of TTP in adults and occurs secondary to anti‐ADAMTS13 autoantibodies. cTTP is the most common form of TTP in children and is responsible for ~5% of adult cases.^3^ cTTP occurs due to recessive mutations in the *ADAMTS13* gene. Whilst a functional ADAMTS13 deficiency is essential for a diagnosis of TTP it is not sufficient to cause disease. Multiple secondary triggers have been identified that initiate disease, these include pregnancy and infection.

### 2.1 Clinical presentation and laboratory findings

In TTP end organ dysfunction often manifests as neurological abnormalities such as headache, confusion, stroke, or seizures. Other common organ involvement includes cardiac and mesenteric ischaemia.^4^, ^5^ In contrast to other forms of TMA (atypical haemolytic uraemic syndrome [aHUS], Shiga toxin‐producing enterohaemorrhagic *Escherichia coli* [STEC]‐HUS described below) renal features are less common. Haematological parameters show the typical presentation of TMA including MAHA and thrombocytopenia, which is often severe <30 × 10^9^/l. Cardiological and neurological symptoms are prognostic of severe disease.^4^

TTP is a medical emergency with a high mortality rate of 90% without treatment.^6^ An ADAMTS13 activity level of <10% is diagnostic of TTP. Although testing ADAMTS13 can be done in a few hours, samples may be sent to expert reference centres, extending the time to obtain results to days.^7^ Rapid commercial kits and automated tests have been developed for faster on‐site testing, but they are not in widespread use.^7^ The PLASMIC score has been developed to assist clinicians in deciding the likelihood of severe ADAMTS13 deficiency while awaiting ADAMTS13 activity levels.^8^ Due to the high mortality of TTP, treatment should be commenced in cases with high clinical suspicion in the absence of ADAMTS13 activity levels. Antibodies can be detected either by ADAMTS‐13 autoantibody assay or inhibitor titres. As non‐inhibitory antibodies, which accelerate the clearance of immune complexes, are clinically relevant, an autoantibody assay is preferential. The inhibitory autoantibodies prevent ADAMTS13 proteolytic activity. False‐negative ADAMTS13 antibody tests occur when the antibody levels are low or when they are highly bound in immune complexes.^9^ ADAMTS13 antigen levels are often varied at presentation but low levels have been associated with poorer outcomes.^4^ During acute presentation it is important to check troponin levels, even in the absence of chest pain, due to high levels of reported ischaemia.

### 2.2 aTTP Treatment

#### 2.2.1 Acute period

The primary treatment for aTTP is plasma exchange (PE) with fresh frozen plasma/Octaplas which remove the autoantibodies and replace ADAMTS13. Plasma exchange is continued until clinical response has been achieved for a minimum of 2 days.^10^ Clinical response is defined by three key parameters: platelet count ≥150 × 10^9^/L, LDH <1.5 times the upper limit of normal and no clinical evidence of new or worsening organ ischaemia.^11^ Many hospitals aim to reduce TTP exacerbations by using a PE tapering technique, over the course of weeks once clinical response has been achieved. The evidence basis for this is limited however^10^ and some authors suggest a higher rate of complications, secondary to PE, in patients on a tapering regime.^12^

Recently caplacizumab has been introduced for the management of patients with TTP. Caplacizumab is an anti‐VWF nanobody, which reduces the adhesion between large VWF multimers and platelets.^13^ The HERCULES and TITAN trials as well as real life outcome data in aTTP patients have demonstrated that the use of caplacizumab, in addition in PE, reduced the time for platelet counts to normalize, the time required on PE and reduced the likelihood of TTP recurrence.^14^, ^15^, ^16^ Acute recurrence, which occurs within 30 days of stopping caplacizumab or PE, is referred to as a clinical exacerbation, recurrence after this time is known as a clinical relapse.^11^ Caplacizumab should be started before PE and then continue daily until 30 days after PE completion.^13^ Caplacizumab is an expensive drug (approximate 270 000 dollars per treatment) and methods to individualize treatment are being investigated, such as alternative day dosing or reducing the initial course.^17^ Volker et al. found that monitoring ADAMTS13 activity allowed clinicians to individualize treatment by shortening or extending caplacizumab duration with successful outcomes.^18^ The study concluded that most aTTP patients could have a shorter duration of caplacizumab treatment but 20% would benefit from a longer duration.^18^ There are huge cost saving implications from these findings, but this method is limited by the amount of time to get ADAMTS13 activity results. Readily available, reliable rapid testing of ADAMTS13 activity would not only allow quick diagnosis but also more individualized, timely treatment. It has been suggested that in some acute cases of aTTP it is possible to treat with caplacizumab without PE^19^ although PE and caplacizumab remain the gold standard.

The third key element of acute aTTP treatment is immunosuppression. Standard immunosuppression is daily prednisolone or pulsed methylprednisolone. Rituximab, a monoclonal antibody directed against the B‐lymphocyte CD20 antigen, is used in aTTP as an additional first line treatment, in refractory cases of TTP and/or to reduce the likelihood of relapse once in remission.

Refractory TTP is used to describe cases where there is no clinical response after five sessions of PE or there is an initial response followed by decline while receiving standard treatment.^11^ Integrated analysis of the TITAN and HERCULES trials demonstrated that caplacizumab reduced the risk of refractory TTP.^15^ In cases of refractory TTP it is important to review the cause of TMA and look for any additional contributing factors to illness such as infection. There are other drugs that have been used in refractory cases, such as vincristine, bortezomib and azathioprine, and splenectomy has also been used.

There is an increased risk of venous thromboembolism (VTE) and arterial thrombosis in aTTP.^20^ Once the platelet count is above 50 × 10^9^/L prophylactic low molecular weight heparin can be used for VTE prophylaxis in addition to compression stockings from initial presentation (if not contraindicated).^21^ Antiplatelet therapy has little evidence base in aTTP and is even more contested due to the higher bleeding risk associated with caplacizumab.^20^ Folate supplementation during active haemolysis is also advised.^22^

#### 2.2.2 Follow‐up period

Following initial presentation patients require regular monitoring of ADAMTS13 activity as well as intermittent treatment such as rituximab to reduce the risk of relapse.

Open ADAMTS13 is a new biomarker of aTTP and can confirm the presence of autoantibodies when there are false negative autoantibody levels due to autoantibody clearance.^23^ In the acute phase of aTTP, ADAMTS13 autoantibodies change the conformation of ADAMTS13 to an open form by binding to the spacer domain.^23^ An IC4 enzyme‐linked immunosorbent assay is used to detect if there is an autoantibody attached at this spacer domain.^23^ Monitoring open ADAMTS13 may allow early detection of subclinical TTP and initiation of prompt treatment.

The Oklahoma TTP‐haemolytic uremic syndrome (HUS) Registry was set up to observe the long‐term outcomes of patients who have had TTP or HUS. Studies using this registry have found much higher rates of cognitive decline and depression in patients who have had TTP. All‐cause mortality was also higher in patients who have had an acute episode of TTP compared to the general population which was not fully accounted for by TTP related deaths.

### 2.3 cTTP Treatment

Unlike aTTP immunosuppression is not required, and management is centred around replacement of deficient ADAMTS13, this is achieved with regular fresh frozen plasma infusions. There is currently a recombinant form of ADAMTS13 in clinical trials, Bax 930, a recombinant ADAMTS13 (NCT03393975).

## 3 COMPLEMENT‐MEDIATED ATYPICAL HAEMOLYTIC URAEMIC SYNDROME

**FIGURE 2:**
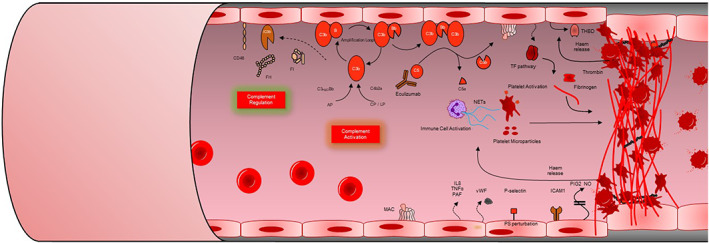
Pathogenesis of complement‐mediated atypical haemolytic uraemic syndrome (aHUS). Complement is activated by the classical (CP) lectin (LP) and alternative (AP) pathways. The AP is a positive amplification loop. C3b interacts with factor B, which is then cleaved by factor D to form the C3 convertase C3bBb. Unchecked this leads to activation of the terminal complement pathway with generation of the membrane attack complex (MAC, C5b‐9) and the anaphylatoxin C5a. Complement regulators including factor H (FH), factor I (FI) and CD46 protect the glomerular endothelium from collateral damage from the AP. In complement‐mediated aHUS, activating mutations in C3 and CFB, loss‐of‐function mutations in CFH, CFI and CD46, and autoantibodies to FH, result in over‐activation of the AP. C5a induces tissue factor (TF) expression endothelial cells and increases tissue plasminogen activator inhibitor −1 in mast cells and basophils. C5a also promotes endothelial cell retraction, exposing underlying basement membrane. Sublytic membrane attack complex (MAC) also induces TF expression and increases adhesion molecule expression on the endothelium (VWF). Downstream effects of sublytic MAC on endothelium additionally include secretion of multimers of endothelial VWF and release of heparan sulphate proteoglycans from endothelial glycocalyx. Complement activation on platelets increases activation markers (CD40L; CD62P) with resulting in activation of the platelets with subsequent release of pro‐thrombogenic TF‐expressing platelet micro‐particles. Haem release from intravascular haemolysis stimulates TF upregulation and Neutrophil extra cellular traps (NETs) formation. NETs released by damaged or activated neutrophils and red cell degradation products have been shown to contribute to thrombus formation. Together these mechanisms lead to immune cell and platelet activation and endothelial cell damage and swelling, with consequent thrombus formation, platelet consumption, vascular occlusion mechanical haemolysis. Eculizumab & ravulizumab binds to C5 to prevent activation of the terminal pathway inhibiting the generation of the effector molecules that cause TMA. Modified from the National Renal Complement Therapeutics Centre 2020/2021 annual report (http://www.atypicalhus.co.uk/)

Haemolytic uraemic syndrome (HUS) is a triad of thrombocytopenia, MAHA, and acute renal failure. Atypical haemolytic uraemic syndrome (aHUS) has been used broadly to describe any case of HUS that is not caused by shiga toxin‐producing bacteria. With the discovery of the multiple underlying causes of aHUS and treatments for them, it has become important to differentiate this term further. In this review we use the term complement‐mediated aHUS to describe aHUS where there is dysregulation of the complement system (Figure 2).

### 3.1 Incidence and pathogenesis

The incidence of complement mediated aHUS in the United Kingdom is 0.42 cases per million per year.^24^ Complement mediated aHUS due to acquired or inherited defects in the alternative pathway (AP) of complement results in excessive solid phase complement activation and ultimately the formation of platelet rich fibrin thrombi, predominantly on renal endothelium. Extra renal manifestations occur in 10%–20% of cases and neurological manifestations are most common.^25^

### 3.2 Complement system overview

The complement system has a fundamental role in the host immune system and consists of >30 interacting proteins which lead to the lysis and opsonisation of pathogens in addition to facilitating the removal of potentially destructive immune complexes and damaged cells.^26^ There are three activation pathways AP, classical (CP) and lectin (LP) which make up the complement system and they all ultimately trigger a shared terminal pathway.^26^

The AP is a constantly turning over positive feedback loop which is recruited by the other pathways to facilitate a rapid response to pathogens and this amplification loop is tightly controlled by a series of cell surface and plasma regulators to prevent over activation.

Complement C3 undergoes spontaneous hydrolysis into C3a and C3b, with C3b attaching to the surface of nearby foreign and host cells. On an activating cell surface (e.g., bacteria), factor B (FB) binds with C3b and is subsequently cleaved by factor D into C3bBb which is the AP C3 convertase. This C3bBb goes onto cleave further C3 into C3a and C3b creating the positive feedback loop. With the addition of further C3b, the C3 convertase is converted into a C5 convertase which cleaves C5 into C5a and C5b which initiates formation of the lytic membrane attack complex (C5b–9). C3a and C5a are anaphylatoxins which induce inflammation.^27^

The CP is activated by molecules such as immune complexes and the LP is triggered when microbial carbohydrates are detected. Both pathways form a different C3 convertase (C4b2a) composed from cleaved complement component C2 and C4.

### 3.3 Regulation

Factor H (FH) is the most important fluid phase regulator of the AP: competing with FB for C3b; accelerating the breakdown of the C3 convertase; and acting as a cofactor for Factor I (FI). Membrane cofactor protein (CD46) is a cell surface complement inhibitor which acts as one of the cofactors to FI in the inactivation of C3b and C4b. FI is the enzyme responsible for complement regulation, inactivating C3b and C4b in the presence of its cofactors (e.g., FH, CD46).

### 3.4 Clinical features and laboratory tests

In complement‐mediated aHUS, AKI is more prominent than in TTP. There is not a rapid screening test for complement‐mediated disease and it is initially a diagnosis of exclusion with genetic and autoimmune analysis subsequently confirming the diagnosis. Complement component levels are routinely tested: C3; C4; FB; FBb; CH50; AH50; FH; FH; C5a, sC5b9; CD46. In aHUS C3, FB, CH50 and AH50 may be low while C5a, C5b9 and Bb may be elevated, however in isolation a complement profile cannot identify nor exclude a complement‐mediated aHUS.^28^

Genetic screening (*CFH*, *CFI*, *C3*, *CFB*, *CD46*, *CFHR1*) is essential to define complement‐mediated aHUS. Mutation screening in aHUS is challenging, because most of the disease‐associated mutations are individually rare, and a significant proportion of variants consist of missense mutations of unknown significance.^29^ In this setting, serum FI, FH and cell surface CD46 levels may help define the pathogenicity of rare genetic variants in these genes. In aHUS >70% of *CFI* and *CD46* rare genetic variants will result in low levels, in *CFH* however, the majority of rare genetic variants result in normal levels of a protein which is non‐functional.^30^ Additionally, the area of the genome in which the *CFH* gene resides arose from several large genomic duplications. These low‐copy repeats can cause genome instability in this region which results in the formation of hybrid genes^31^, ^32^, ^33^, ^34^, ^35^, ^36^ which require copy number analysis to detect in addition to standard sequencing.

Screening for autoantibodies to FH should be performed with epitope mapping studies to the C terminus of the protein suggestive of aHUS. Although autoantibodies to FI have been demonstrated in complement‐mediated aHUS their role in pathogenesis is yet to be confirmed in replication studies.^37^

In addition to genetic screening for aHUS associated complement genes, other genetically mediated TMA should be screened for (e.g., diacylglycerol kinase epsilon [DGKE], MMACHC).

### 3.5 Genetic complement‐mediated aHUS

Complement‐mediated aHUS can be caused by genetic mutations and autoantibodies. Factor H mutations are the most frequent accounting for ~25% of cases and predominate in the C‐terminus of the protein preventing cell surface complement regulation.^38^ Loss‐of‐function mutations have also been described in the regulators *Factor I* ^39^ and *CD46*.^40^ Pathogenic activating mutations in *C3* ^41^ and complement factor B have also been described.

### 3.6 Predisposition, penetrance, and triggers

The genetic variants reported in complement‐mediated aHUS are not causative but are predisposing with penetrance low. Complement‐mediated aHUS can develop throughout life but there is an age‐related penetrance with the likelihood of disease development increasing in those who have more than one complement mutation. Single nucleotide polymorphisms (SNPs) have also been shown to alter penetrance.^25^

Most cases of complement‐mediated aHUS require an environmental trigger, which is thought to initiate a complement cascade that reveals the latent regulatory defect. Studies have shown that infectious events such as gastroenteritis and respiratory tract infections trigger complement mediated aHUS in about 50% of cases.^42^, ^43^ Other common triggers are pregnancy and drugs.^44^

### 3.7 Acquired complement‐mediated aHUS

FH antibody mediated disease mostly presents in childhood and often is preceded by gastrointestinal symptoms.^45^ There is an association between the development of FH antibody mediated aHUS and homozygous deletion of *CFHR3* and *CFHR1*, but the underlying mechanism is not understood.^46^ Although factor I antibodies have been identified, they are rare and their impact on disease generation is unknown.

### 3.8 Treatment

Before the development of complement inhibitors PE was the main treatment for complement‐mediated aHUS. The removal of FH antibodies and overactive complement components with replacement of defective complement regulatory proteins made PE a logical treatment. PE is started, in adults, presenting with a TMA, while waiting for the ADAMTS13 activity level to exclude TTP. In children, TTP is rare, and PE has a more side effects, therefore, complement inhibitors are first line.^47^ PE remains the only treatment in countries where access to complement inhibitors is restricted due to cost.

There were historically poor outcomes with PE treatment alone. There were high rates of end stage renal failure (ESRF) or death at 3–5 years (36%–48% in children and 64%–67% in adults).^48^ Prognosis varied pre complement inhibitors depending on the genetic mutation, *CD46* mutations generally had the best outcomes and *CFH* mutations had the worst.^42^, ^48^

Renal transplantation also had poor outcomes in aHUS with recurrence in 60%–70% of patients, most in the first year after transplant.^49^ Prognosis following transplantation was again predicted by mutation type as individuals with isolated mutations in CD46 had a low risk of recurrence.

#### 3.8.1 Eculizumab

Eculizumab was the first targeted treatment for aHUS. It is a recombinant humanized monoclonal antibody to the C5 complement protein, preventing the cleavage of C5 into C5a and C5b.^50^ It has revolutionized the treatment of patients with complement‐mediated aHUS and is also thought to be safe in pregnancy.

Those receiving treatment with eculizumab are particularly vulnerable to infection with encapsulated organisms as host defence is dependent on the complement membrane attack complex. Therefore, vaccination for *Neisseria meningitides* is required as well as prophylactic antibiotics for all those taking eculizumab.^24^

#### 3.8.2 Eculizumab nonresponse

There are cases of non‐response to eculizumab in TMAs. Polymorphisms in C5 have been identified (pR885H;pR885C) which prevent eculizumab binding to C5 and therefore prevent its function to block the terminal pathway. In these patients' plasma exchange is recommended. Additionally, some non‐response is due to TMAs with mutations in non‐complement genes, for example, *MMACHC*, *DGKE*,^51^ *INF2*.^52^

#### 3.8.3 Ravulizumab

Ravulizumab was the second agent approved by the European Medicines Agency and US Food and Drug Administration, for the treatment of patients with aHUS. Ravulizumab was engineered from eculizumab and targets the same epitope in C5. A histidine switch was performed in the complementarity‐determining regions of eculizumab to preserve binding to C5 in serum but to allow dissociation of C5 from ravulizumab in the acidified endosome. Additionally amino acid alterations to the Fc region of eculizumab resulted in increased efficiency of neonatal Fc receptor‐mediated recycling. This resulted in ravulizumab having an increased half‐life of ~52 days compared to ~11 days with eculizumab and therefore, up to an 8‐week dosing interval with ravulizumab versus 2 weekly with eculizumab.

There has not been a direct comparison of ravulizumab to eculizumab in aHUS with both the adult and paediatric studies being single arm studies.^53^, ^54^, ^55^ This has led to concerns of equivalent efficacy with more deaths and fewer patients stopping dialysis in the adult ravulizumab trial compared to the historic eculizumab trials.^56^ The very low mutation/factor H autoantibody rate in this trial suggests that many patients enrolled did not have complement‐mediated aHUS. Indeed, the granular data presented around the deaths in these studies demonstrated that they were not complement‐mediated aHUS (e.g., sepsis).^54^, ^55^ Reassuringly in the paediatric study, where the differential diagnosis of complement‐mediated aHUS is less broad, the mutation/factor H autoantibody rate was higher and 94.4% of patients had a complete TMA response by 50 weeks.^53^

#### 3.8.4 Disease driven versus continuous eculizumab therapy

Eculizumab was licensed for long term treatment of aHUS however there is no evidence for this recommendation. Indeed, there was reason to believe that continuous therapy may not be necessary. In those with pathogenic mutations in the complement system there is a high degree of non‐penetrance with a trigger necessary for disease onset, often well into the adult years. Additionally, in the era of PE for aHUS, treatment was routinely withdrawn with only a proportion relapsing and requiring ongoing PE. Eculizumab has changed the natural history of the disease and many patients who would previously have reached ESRF despite plasma exchange may remain dialysis free and susceptible to disease relapse.

Recently a succession of case reports and series has reported on the outcome of eculizumab withdrawal.^24^, ^57^, ^58^, ^59^ Unsurprisingly a proportion of patients experienced relapses after withdrawal, however, close monitoring with reintroduction of eculizumab rapidly controlled the TMA with return of renal function to baseline. Although confounded by their non‐prospective basis and likely clinical selection bias, analysis of these reports has suggested an intermittent disease driven regime is possible at least in some patients. It is interesting to note that relapse was almost exclusively seen in those with a pathogenic mutations, although conversely not all individuals with mutations relapsed.

The first of a series of 3 prospective studies investigating eculizumab withdrawal in aHUS, the STOPECU study, has recently reported suggesting that eculizumab withdrawal can be undertaken.^60^ In this study, aHUS relapse was only seen in those individuals with an underlying complement gene mutation. Female gender and an increased sC5b9 level at eculizumab withdrawal was associated with risk of recurrence. Of 13 patients who had relapsing aHUS and were retreated with eculizumab, 2 had worse renal function with 1 of these requiring a renal transplant. The remaining prospecting trials SETSaHUS (United Kingdom) and CUREiHUS (Holland) will further guide practice. It is likely that a personalized approach to cessation will ultimately be adopted, driven largely by the presence and type of mutation, the degree of residual renal function and whether there is a renal transplant. A critical feature for an intermittent treatment regime will be determining the optimal monitoring strategy with urinalysis, blood pressure monitoring, and blood testing being variably used currently.

## 4 OTHER GENETIC TMAs

### 4.1 Diacylglycerol kinase epsilon TMA

This is a rare form of aHUS (0.009/million/year in the United Kingdom^51^) which typically presents in children under 2 years. It was first reported in 2013.^61^ Recessive mutations in *DGKE* have genetic pleiotropy resulting in an aHUS or mesangioproliferative glomerulonephritis pathology. Clinically, patients present with similar haematological and biochemical features as complement‐mediated aHUS, with the notable addition of heavy proteinuria.^51^ Occasionally cases can present after a viral trigger or prodromal diarrhoea.

Although the exact mechanism behind DGKE induced aHUS remains under investigation, it has been demonstrated that a complex array of autocrine signalling events downstream of VEGFR2 that are mediated by PGE2 result in endothelial activation and a thrombogenic state.^62^

No treatment has been found to be consistently superior to supportive measures and relapses within the first few years of life are common. A proportion of these patients progress to ESRF with no recurrence post transplantation documented.^51^

### 4.2 Cobalamin C aHUS

Cobalamin C (vitamin B12) type methylmalmonic aciduria and homocystinuria is a disease of vitamin B metabolism that presents predominantly in childhood. It is due to recessive mutations in *MMACHC*.^63^ Plasma homocysteine and plasma and urine methylmalonic acid level are elevated in MMACHC mediated disease. MMACHC enables cobalamin to be converted to its active forms MeCbl and AdoCbl. Reduction in MeCbl and AdoCbl results in failure to convert homocysteine to methionine and methylmalonyl‐CoA to succinyl‐CoA, respectively.^64^

Presentation with methylmalmonic aciduria and homocystinuria Cobalamin C type is generally with neurological, cardiac, ophthalmic, and developmental abnormalities.^63^ A proportion of these patients also develop a TMA and the mechanism behind this is not fully understood.^63^ It is thought to relate to the raised homocysteine levels damaging the endothelium of the kidney. Treatment is with hydroxycobalamin and betaine. No benefit has been shown from complement inhibiting therapy.^65^

### 4.3 Infection associated TMAs

#### 4.3.1 Shiga toxin‐producing enterohaemorrhagic *Escherichia coli*‐HUS

STEC‐HUS is by far the most common form of TMA, mainly occurring in children under 5 years of age, and accounts for 90% of the cases of HUS in children. STEC‐HUS occurs following infection with the STEC, which is found in the intestines of healthy animals such as cattle. The main transmission routes are through undercooked meat, unpasteurised dairy produce, and direct animal contact, but other routes occur, such as ingestion of contaminated vegetables.^66^ Patients typically develop bloody diarrhoea, abdominal pain and vomiting 3 days after exposure to the STEC. In the majority of children this is self‐limiting, however 10%–15% of children will go onto to develop HUS 7–10 days after symptom onset.^66^

Once STEC is in the intestines, it attaches to the epithelium where it produces Shiga toxin which translocates through to the blood stream. The Shiga toxin travels through the vasculature attached to blood cells such as leucocytes and platelets. When it encounters and attaches to the Gb3 receptors, which are present in the kidneys, brain and gut, it is transported into the cell via endocytosis.^67^ Once inside cells it results in cell death via inhibiting protein synthesis and ribosomal function.^1^ The predominant presentation in children has been hypothesized to be due to the prevalence of anti‐Shiga antibodies in adults and/or the increased glomerular expression of the Gb3 receptor in children.

It may be initially difficult to differentiate STEC HUS from complement‐mediated aHUS as almost a third of patients with complement‐mediated aHUS have concurrent diarrhoea or gastroenteritis and ~5% of those with STEC‐HUS have no prodromal diarrhoea.^47^ Therefore, faecal culture, to detect *E. coli* O157:H7 and faecal PCR for STEC genes should be performed to identify this form of disease in all individuals presenting with HUS.

STEC HUS is usually a self‐resolving illness and most recover within a few weeks with supportive measures such as fluid resuscitation, dialysis and red cell transfusion but 30% of patients go onto have long term renal complications. There are no specific treatments for STEC‐HUS. Although complement activation is seen during STEC HUS the role of complement inhibitors as treatment is still debated due to the lack of controlled data.^67^ Trials investigating the role of eculizumab in the treatment of STEC have yet to report (ECUSTEC ISRCTN89553116, NCT02205541).

#### 4.3.2 *Shigella dysenteriae* type 1

Shiga toxin was first detected in *Shigella dysenteriae* and this remains an important cause of HUS in developing countries. The clinical features are similar to STEC‐HUS but the diarrhoea is initially more watery before becoming bloody or mucoid and fever is common. HUS develops at ~7 days after the diarrhoea has resolved. Although less than 10% of children with the infection will develop a TMA, it has a high mortality rate and those who survive are more likely to develop chronic kidney disease.

#### 4.3.3 Pneumococcal HUS

Invasive infection with *Streptococcus pneumoniae* may cause a TMA. Most cases present with pneumonia (often with empyema) but it has also been reported with meningitis or sinus/ear infections. Patients presenting with pneumococcal HUS (pHUS) are unwell and frequently require intensive care treatment.^68^ Three quarters of children who develop pHUS require dialysis and a third go on to develop ESRD.

The development of HUS is thought to be due to *S. pneumoniae* producing neuraminidase.^69^ This sialidase cleaves sialic acid residues from glycoproteins on endothelial cell membranes, platelets and erythrocytes. This results in the exposure of a cryptic T antigen (Thomsen–Freidenreich antigen) which is bound by pre‐formed circulating IgM antibodies resulting in platelet aggregation, endothelial cell damage and ultimately TMA. This leads to a positive Coombs test in contrast to other forms of TMA. It has also been suggested that FH binding may be reduced by the cleavage of sialic acid leading to impaired complement regulation and endothelial damage.^70^

Treatment is supportive (dialysis, red cell transfusion) and treatment of the underlying infection. PE is a contentious issue due to concerns that infusing plasma containing more anti‐T IgM will worsen the disease state. There are case studies using washed/plasma reduced blood components such as albumin with PE with good results which is thought to be due to removal of host anti‐T IgM and the neuraminidase.^71^ Mortality remains high at up to 10%.

#### 4.3.4 Human immunodeficiency virus

The mechanism by which human immunodeficiency virus (HIV) causes aHUS is unclear but is thought to be due to endothelial damage. The incidence of TMA in patients with HIV has reduced significantly since the application of highly active antiretroviral therapy and is now ~0.3%.^72^ Treatment is supportive with optimisation of antiretroviral therapy.

#### 4.3.5 Covid‐19 infection

As with many infections, severe acute respiratory syndrome‐corona virus‐2 (SARS‐CoV2) has been described as a potential trigger of complement‐mediated aHUS.^73^ Marked complement activation is seen in COVID‐19 with sC5b9 correlating with disease severity,^74^ with marked endothelial cell injury is seen.^75^ Thus, in those with a latent complement regulatory defect, this complement stimulus is sufficient to provoke complement‐mediated aHUS. In the setting of COVID‐19 induced complement‐mediated aHUS, C5 inhibition would be the treatment of choice.

Additionally, complications of acute infection have been described including macro thrombosis and AKI. Almost a third of patients can still develop thrombosis despite anticoagulation in this prothrombotic state.^76^ While C5 inhibition was proposed for to prevent these broader thrombotic events, a trial of ravulizumab in COVID‐19 was stopped due to lack of efficacy.

#### 4.3.6 Other infections

As seen with COVID‐19, many viral, bacterial, and parasitic diseases have been suggested as triggers of complement‐mediated aHUS.^30^ One study looking at genetic mutations in complement‐mediated aHUS found that 73% of patients had an infectious trigger.^43^

### 4.4 Pregnancy associated TMA

Pregnancy and the postpartum are high‐risk periods for TTP and complement‐mediated aHUS.

Pregnancy related TTP typically presents in the second and third trimesters. Pregnant women account for up to a third of adult presentations of TTP with approximately a quarter of these being cTTP (cf. 5% in adult‐onset TTP).^77^ The underlying cause for this is thought to be due to the increase in VWF production, which is common for all pregnancies, which subsequently increases consumption of ADAMTS13. In those with genetic mutations, this additional consumption of ADAMTS13 can lead to levels low enough for TTP to develop.^77^ There is a high mortality risk to the mother and the foetus and PE should be instituted urgently. The PLASMIC score has not yet been validated in pregnancy.

The experience of caplacizumab in pregnancy is, as yet, limited to case reports and safety is unknown.^78^ There have been foetal abnormalities reported with rituximab and therefore use during pregnancy is with caution.^77^

The risk of relapse in subsequent pregnancies is high. In subsequent cTTP pregnancies relapse is the rule without regular FFP infusions.^77^ In aTTP the relapse rate in pregnancy has been 50% and there is an aim to have good ADAMTS13 activity and low antibody levels prior to conception to prevent relapse.^79^ Rituximab has been used successfully to increase ADAMTS13 activity levels in this patient group, but it is advised to wait at least 6 months before conception after completion of therapy.^79^

In contrast to TTP, in pregnancy, aHUS tends to occur in the postpartum period. Studies have shown that at least 50% of women who develop HUS as a result of pregnancy, have complement mutations, which is similar to the general population presenting with aHUS.^44^ Pregnancy is therefore thought of as an aHUS trigger and can be treated successfully and safely with eculizumab.

Pre‐eclampsia, eclampsia, and haemolysis elevated liver enzymes low platelets (HELLP) syndrome are part of a spectrum of pregnancy conditions that are part of the differential diagnosis of TTP and aHUS.^77^ Pre‐eclampsia is characterized by hypertension and proteinuria. HELLP is a more severe form of the disease spectrum, which can lead to a glomerular endotheliosis. The pathogenesis of these conditions is not fully understood. There is known endothelial dysfunction thought to be due to greater circulating levels of the soluble form of the vascular endothelial growth factor receptor (sFlt‐1), syncytiotrophoblast‐derived antiangiogenic factors and soluble endoglin. Although 8%–10% of patients with pre‐eclampsia and HELLP have been found to have complement gene rare variants, most were not thought to be pathogenic.^80^ Although mouse models of pre‐eclampsia and elevated complement plasma levels in patients has suggested a role of complement, pre‐eclampsia has occurred in women taking eculizumab for paroxysmal nocturnal haemoglobinuria and aHUS, suggesting the terminal pathway of complement is not critical for disease to manifest.

Diagnostic tests include assessment of blood pressure and proteinuria, liver enzymes which are raised in HELLP and conducting sFlt‐1/PIGF ratios to assess likelihood of hypertensive complications in pregnancy. Ratios indicative of pre‐eclampsia, eclampsia or (HELLP) would be 110 after 34 weeks gestation and 85 before 34 weeks gestation.^77^

As TTP, aHUS, HELLP and other TMA mimicking pregnancy conditions are life threatening to the mother and the foetus, rapid commencement of correct management is key to patient survival and good outcomes. Guidelines for the assessment and management of TMAs in pregnancy have been developed by an international multidisciplinary working group.^77^

### 4.5 Drug induced TMA

Many drugs have been associated with TMAs but there are only a few where causality has been established. Two main mechanisms of drug induced TMA are described: immune mediated damage and direct toxicity. Quinine therapy can lead to the development of autoantibodies to platelet glycoproteins Ib/IX and/or IIb/IIa complexes resulting in a TMA.^81^ Drugs that have been proved to cause a TMA via direct toxicity include interferon β^82^ and bevacizumab,^83^ although chemotherapeutic agents (e.g., gemcitabine) and immunosuppressive agents (e.g., calcineurin inhibitors, ciclosporin and tacrolimus) may also be implicated. The treatment recommendation is cessation of the suspected drug and supportive care. Ticlopidine can lead to the development of TTP associated with ADAMTS13 antibodies.^84^ Treatment in this case is as per TTP and the withdrawal of ticlopidine.

### 4.6 De novo TMA post solid organ transplant

The development of de novo TMAs have been reported in patients after solid organ transplants.^85^ The pathogenesis of these de novo TMAs is thought to be multifactorial with antibody mediated rejection, ischaemia reperfusion injury, calcineurin inhibitors and infections leading to endothelial damage and subsequently triggering a TMA. In a study of patients who had received a renal transplant and later developed a TMA, 29% were found to have underlying complement mutations.^86^ Treatment involves reviewing and altering immunosuppression regimes, treating underlying viral infections, and the use of complement inhibitor therapy if complement‐mediated aHUS is felt likely.

### 4.7 Bone marrow transplant TMA

TMAs can occur as a complication of bone marrow transplant and have a high mortality rate.^65^ As with solid organ transplantation the underlying aetiology is myriad. It is hypothesized that a vascular form of graft versus host disease (GVHD) may lead to this type of TMA due to the association with high grades of GVHD in these patients.^87^ There is some evidence of complement activation and rarely functionally significant mutations in genes described in aHUS have been reported.^87^ Eculizumab treatment has been reported however prospective trial outcomes are awaited.^88^

### 4.8 Malignancy associated TMA

TMAs related to malignancy can either be a direct consequence of the malignancy or the chemotherapy agents used to treat it and it is generally difficult to differentiate between the two. It has been hypothesized that in disseminated malignancy, embolic tumour cells in the microvascular may cause direct erythrocyte shearing.^89^ Treatment is supportive and includes discontinuation of chemotherapy agents although the prognosis is poor due to the underlying malignancy.

### 4.9 Autoimmune TMA

In systemic lupus erythematosus (SLE), scleroderma renal crisis (SRC) or catastrophic antiphospholipid syndrome (CAPS) presentations with TMA are well recognized although the mechanisms are unclear.

In SLE the CP is a key driver of disease with AP recruitment also seen, although to date there is no correlation with complement activity and the development of a TMA in SLE. Case reports describe the use of eculizumab in SLE‐TMA suggested a response^65^ however larger case series failed to replicate this. Currently standard immunosuppression remains the treatment of choice.

Approximately 30% of CAPS cases result in a renal TMA with an overall CAPS morality rate of 36%.^90^ In CAPS, triple therapy with glucocorticoids, anticoagulants as well as PE and/or intravenous immunoglobulins has been shown to reduce mortality compared to other combinations.^91^ There is evidence of complement activation in CAPS (in human and mouse studies), as well as case reports of a good outcomes after eculizumab use.^65^

There is little evidence for the role of complement in SRC. It occurs in ~10% of patients with systemic sclerosis, and TMA manifests in 45%–50%. Since the introduction of ACE‐inhibitors as the treatment for SRC, mortality rates of SRC have reduced significantly.^92^

### 4.10 TMA associated with glomerular disease

Histopathological changes of TMA have been described in number of glomerular diseases without concurrent MAHA or thrombocytopenia, for example, IgA, ANCA associated vasculitis, membranous nephropathy, focal segmental glomerulosclerosis and C3 glomerulopathy (C3G)/mesangioproliferative glomerulonephritis (MPGN).^52^, ^65^, ^93^

Although C3G/MPGN are complement‐mediated diseases the pathogenesis is slightly different to aHUS. In C3G autoantibodies and genetic mutations result in fluid phase complement activation^94^ compared to the solid phase cell surface activation seem in aHUS.^38^ Concurrent and sequential occurrences of C3G and TMA have been reported.^95^

### 4.11 Hypertensive TMA

Severe hypertension can present with both pathological evidence of TMA and clinical features (AKI, MAHA and thrombocytopenia). This can be indistinguishable from a complement‐mediated TMA where the majority of patients will have severe hypertension. This has been demonstrated in a retrospective study reviewing nine patients misdiagnosed with clinical hypertensive TMA, who develop ESKF despite blood pressure control. Genetic analysis found that eight had rare variants in complement genes in keeping with complement‐mediated aHUS.^79^ Pragmatically failure of BP control and supportive management to control MAHA and thrombocytopenia will often result the initiation of PE or eculizumab until complement evaluation is available. In hypertensive TMA a large proportion will develop ESKF and genetic analysis should be undertaken prior to listing for renal transplantation.^47^

### 4.12 Summary

TMAs can manifest in an array of clinical presentations. Recent research developments have revolutionized the understanding of the disease processes and lead to the development targeted therapeutics for TMAs. These therapies have markedly improved morbidity and mortality from disease.

## CONFLICT OF INTEREST

David Kavanagh has received consultancy income from Gyroscope Therapeutics, Silence Therapeutics, Alexion Pharmaceuticals, Novartis, Apellis, and Sarepta.

## Data Availability

Data sharing is not applicable to this article as no new data were created or analyzed in this study.
